# The Story of the Insane from Year to Year

**Published:** 1891-08-22

**Authors:** 


### The Story of the Insane from Year to Year.

(Fourth Series.)

### KENT COUNTY ASYLUM, BARMING HEATH.

Here the numbers have gone down from 1,438 on the first of January, 1890, to 1,420 on the last day of the year; the decrease being explained by the fact that some of the unions formerly sending patients to Barming Heath are now included in the County of London.

The admissions reached 254, the recoveries 124, and the deaths 122. Put into percentages we find that the recoveries stand at 48·8, and the deaths 8·4, the first being decidedly above the average and the last somewhat below it. Reference to Table V. tells us that phthisis killed 33 patients, brain diseases 49, and enteritis 2.

Dr. Davies says, and we agree with him, that a report such as he has to present "should not be the medium for expressing opinions on medical topics." Considerable extention of leave of absence to attendants has been given in this asylum. They get one day every week in addition to fourteen days' annual leave. We are surprised to hear that this liberal arrangement has not worked well, and Dr. Davis cannot recommend other asylums to follow the example.

Once more we have the Lunacy Act severely handled, but we feel bound to admit not more severely than it deserves. "The New Lunacy Act has entailed excessive labour upon the staff, labour out of all character with the benefit supposed to be derived from the vexatious enactments it contains. It is a pity that in drafting such an Act all practical utility should have been ignored. It bears evidence of a want of knowledge in almost every clause."

The visitors do not publish their report, and we did not see any statement of the weekly cost per head.

### THE EXETER CITY ASYLUM.

The numbers in this asylum rose during the year from 208 to 328, but only 9 of the increase came from the city of Exeter, the rest being chargeable to London and Somerset. The admissions were 196, the recoveries 23, and the deaths 15. Excluding the transfers, the recoveries are 38·3 on the admissions, and the deaths 5·2 on the average number resident. Forty-five of the admissions owe their insanity either to drink or to hereditary tendency. There were only two deaths from phthisis, but pneumonia accounted for four, and bronchitis for one. There cannot be a doubt that cheerful and bright surroundings tend to promote the cure of the curable, and to increase the feelings of contentment of the incurable. Dr. Rutherford recognised this fact, and he tells us "that many improvements have been made in the wards and the asylum generally, every endeavour being made to brighten the patient's surroundings and make them as comfortable as possible. . . No distinction is made between the wards, whether occupied by the best or the worst class of patients—as none are ever reduced so low as to be incapable of some measure of improvement; but if their surroundings were gloomy, and if they were treated as though their improvement were considered hopeless, no improvement could be expected, and it would be impossible to arouse in them anything allied to self-respect." Exactly. The element of hope must be ever present to the asylum physician, and despair must be banished from his mind.

The Visiting Committee "are of opinion that the whole management of the institution reflects the very highest credit upon Dr. Rutherford and those working under him." We did not notice any statement of the weekly cost of maintenance. Why not publish it?

